# Stability of infants’ preference for prosocial others: Implications for research based on single-choice paradigms

**DOI:** 10.1371/journal.pone.0178818

**Published:** 2017-06-02

**Authors:** Tyler Nighbor, Carolynn Kohn, Matthew Normand, Henry Schlinger

**Affiliations:** 1 Department of Psychiatry, University of Vermont, Burlington, Vermont, United States of America; 2 Department of Psychology, University of the Pacific, Stockton, California, United States of America; 3 Department of Psychology, California State University, Los Angeles, California, United States of America; University of Portsmouth, UNITED KINGDOM

## Abstract

Some research suggests infants display a tendency to judge others’ prosocial behavior, and in particular, that infants show a strong preference for prosocial others. For example, data from one frequently cited and well-publicized study showed that, after watching a puppet show with three puppets, 74% of infants chose the puppet that “helped” rather than the puppet that “hindered” a third puppet from attaining its goal. The purpose of the current investigation was to replicate these methods and extend them by including a within-subject measure of infant puppet choice across repeated trials to assess the stability of infants’ choice. In the current study, 20 infants viewed a puppet show and chose between two puppets (i.e., helper or hinderer) immediately following the puppet show. Although results were similar to previously published work on the first-choice trial (65% of infants chose the helper puppet on the first trial), infants did not consistently choose the helper across trials; several infants demonstrated a side preference, with 9 infants almost exclusively choosing puppets presented on the right or left side. The current investigation addressed limitations of previous research by including a between-subjects (replication) as well as a within-subjects (extension) repeated measure of choice that allowed for the examination of the stability of the choice measure. Our results, particularly in light of other failed replications, raise questions regarding the robustness of infants’ preference for prosocial others and the reliability and validity of the single-choice paradigm.

## Introduction

A recent line of research suggests infants as young as 5 months old [1–5] and older infants and toddlers [6–8] are capable of socially evaluating others’ behavior, and show a strong preference for prosocial others. In a typical experimental arrangement, an infant first views a staged scene in which a focal puppet attempts to do something such as climb a hill or open a box containing a toy. Two other puppets, a “helper” and a “hinderer,” alternately interact with the focal puppet in distinct ways. The helper puppet assists the focal puppet with the task at hand, and the hinderer puppet interferes with the same task. For example, as the focal puppet attempts to open the lid of a box, the helper puppet moves in and helps lift the lid open [2]. In contrast, on a different trial the hinderer moves in and forcefully closes the lid. Variations on this basic scenario include colored shapes with eyes trying to climb a hill and being helped or hindered by other shapes [1]. After watching one of these scenarios, the experimenter presents both puppets (helper and hinderer) to the infant seated in his or her parent’s lap. The first puppet the infant selects (typically defined as concurrently looking at and touching the puppet) is considered to be the infant’s preferred puppet. Infants who choose the “helper” puppet are described as preferring the prosocial other.

Although infants’ preference for prosocial behavior is an interesting possibility, failed replications [9,10] suggest several features of the experimental arrangement warrant attention before clear conclusions can be drawn. Many things happen in the staged scenario, and the putative prosocial element is only one of them. For example, using similar, though not identical, methods, Scarf et al. [9] found that the puppet movements of bouncing and colliding resulted in more infants choosing the puppet that bounced, irrespective of its helper or hinderer status. Salvadori et al. [10] directly replicated the methods of Hamlin and Wynn [2] with infants aged 8 to 9 months and found that only 15 of 24 infants (62.5%) selected the helper puppet. Even following subsequent procedural modifications suggested by Hamlin ([10]), only 12 of 24 (50%) infants selected the helper in their second experiment. However, 17 of 24 (70.8%) infants selected the puppet presented on the right side, indicating that something aside from the social aspect of the puppet show might direct infants’ choices. Cowell and Decety also replicated Hamlin et al.’s [1] puppet paradigm, and in their study only 54 (50%) of infants chose the helper over the hinderer [11]; unfortunately, the authors did not report information about infants’ side preferences. Together, these studies suggest that factors other than a preference for prosocial others, such as a preference for the side on which a puppet is presented, might influence infants’ choices.

If infants have a preference for prosocial others, and if this preference can be assessed using a puppet show immediately followed by a puppet preference assessment (choice-trial), infants’ prosocial preference should be relatively stable within a single session and therefore observable across consecutive choice-trials within a short time frame. If the effect is indeed robust, then the between-subject variability in previous studies [10,11] poses a problem. There is a substantial body of literature on the use of preference assessments, including those for typically developing toddlers [12] and for individuals who cannot otherwise communicate their preferences [13,14]. These preference assessments involve presenting stimuli over multiple trials and, importantly, counterbalancing the placement of items both between and within subjects to determine the most and least preferred items, often in terms of a relative preference hierarchy. Thus, explicit attempts are made to control for side bias. Preference is determined, even among nonverbal individuals, by examining responding across multiple trials, not from any individual trial. Although preferences can change over time [14–16], individuals generally select items deemed preferred much more frequently than less preferred items within a single session, yielding a hierarchy of most to least preferred [13, 14, 15]. Given that infants tend to engage in perseverative reaching [17, 18] and that Salvadori et al. [10] found over two-thirds of infants reached for the right side, studies using the infant puppet choice paradigm ought to carefully examine and control for side preferences.

The purpose of the current investigation was to replicate the methods of Hamlin and Wynn [2] while also employing a within-subject repeated-measures design with four additional choice measures for each infant, permitting an assessment of the stability of infants’ choices without compromising the original one-choice methodology used by Hamlin and Wynn [2].

## Materials and methods

### Participants

University of the Pacific human subjects institutional review board approved all aspects of this study (IRB Protocol #14–31) prior to the start of data collection. We reviewed all study procedures with each parent participant and obtained their written consent, including consent to video record, prior to their enrollment in the study; infants participated only after their parents provided consent. Twenty healthy, typically developing infants, ages 5 to 16 months (*M* = 8.9, *SD* = 3.46) and their parents participated. Although Hamlin and Wynn [2] used groups of 5 and 9 month olds, researchers have used infants aged 10 to 23 months to examine similar questions using comparable methodologies and garnered similar results (15-16-month-olds [5, 11]; 10-month-olds [1,9]; 19–23 month-olds [5]; 12-month-olds [19]). In addition to examining the infants as a single group, we also separated them into two groups by age for specific analyses (described below); one group of 5–8 month-old (*n* = 11; *M* = 6.18, *SD* = 1.25) and a second group of 10–16 month-old (*n* = 9; *M* = 12.11, *SD* = 2.42). Based on recommendations concerning data collection and analysis [20], prior to the start of data collection we determined we would obtain data from 20 infant-parent dyads and conduct five choice trials with each infant. Participants were recruited via flyers and word of mouth. Each parent was provided a $10 gift card as compensation for participation.

### Materials

All puppet shows took place inside a 122 cm wide and 66 cm high display, the same dimensions used by Hamlin and Wynn [2]. The display was placed approximately 163 cm away from the chair where the infant and parent were seated (see S1 Fig). A curtain was attached to the front of the display, which could be lowered to hide the display and raised to reveal the display (see S2 Fig). The puppets, a yellow duck puppet and two identical gray elephants (one in a red shirt, the other in a yellow shirt) were used because Hamlin et al. [5] provided supplementary videos online, enabling the use of nearly identical puppets. The clear box and brightly colored rattle were similar to those used by Hamlin and Wynn [2]. All sessions were video recorded.

### Counterbalancing

The shirt color of puppets, order of scenarios during habituation, the side occupied by the two puppets during habituation, and the side placement of puppets during the choice measure were counterbalanced across participants. Additionally, presentation of the two puppets was counterbalanced for each subject across repeated choice trials.

### Response measurement

Our definition of infant choice was identical to Hamlin and Wynn’s [2]: the first puppet the infant looked at and concurrently touched (p. 33). If the infant touched but did not look at the puppet or looked at a puppet without touching it, no choice was scored and a new trial began. If the infant did not reach for a puppet within 10 s, a new trial began and the puppets were presented again. This occurred a total of three times: during one trial for one participant, and during two trials for two participants.

### Procedure

#### Familiarization process

Each infant was seated on his or her parent’s lap in front of the stage (S1 Fig) and the experimenter presented a clear box, opened the box, and removed and shook the rattle in the box in front of the infant, all of which lasted approximately 20 s; infants viewed the familiarization processes twice, as described by Hamlin and Wynn [2].

#### Habituation trials

Following the familiarization process, infants viewed the habituation trials during which the helping and hindering puppet shows were presented on alternating trials. Each infant’s looking time during each trial was measured, and each infant viewed puppet shows until looking times reached the pre-set criterion used by Hamlin and Wynn [2] as described by Hamlin et al. [1]. During habituation trials, the two elephant puppets were placed at the back corners of the stage, equidistant from each other and from the infant. The box containing the rattle was placed in the center of the stage equidistant from each elephant puppet. An experimenter, not visible to the parent or the infant, performed the puppet show while wearing long black gloves and placing his hands through a black curtain at the back of the stage. Prior to viewing the scenarios, parents were instructed to sit quietly with their infants and not to direct their infant’s attention in any way; infants were shown the puppet shows in alternating sequence until either the sum of the looking times on 3 consecutive trials after the first 3 trials was less than half of the sum of the looking times on the first 3 trials, or until 14 trials had elapsed [1–2]. On average, infants required 10 habituation trials (Range of 6–14 trials).

We carefully reviewed Hamlin et al.’s [5] online supplementary videos in an attempt to replicate procedures not described in their published papers (e.g., approximate duration of each puppet’s action, exact puppet movements) and to address their critique of Scarf et al.’s [9] lack of attention to specific puppet show details. The study methods described below are based on this analysis of the supplemental videos. Table 1 lists methods used in both the helper and the hinderer puppet shows. Differences between the two puppet shows are described under the subheadings entitled *Helping event puppet show* and *Hindering event puppet show* below.

**Table 1:** Methods used in both the helper and the hinderer puppet shows.

• Events (i.e., a single puppet show trial) lasted approximately 15 s.
• At the start of each trial, the experimenter stated, “Up goes the curtain!”
• Following that, the protagonist (duck) puppet appeared from the back center of the stage, paused for approximately 1 s behind the center of the box, and moved to one side of the box. The side from which the protagonist entered was consistently opposite that of the helper or hinderer character.
• The duck moved, left foot followed by right foot, to either the left or right side of the box, and then slid briefly (0.5 s) towards the front of the box. The side of the box to which the duck was moved was counterbalanced across trials.
• The duck’s entire body twisted to look at the box (head moved towards box while bottom remained stationary), and then sat straight up and faced the infant. This occurred twice.
• Next, the duck was positioned on the corner of the box (either right or left), faced down, and the duck lifted the lid of the box.
• On the first attempt, the duck lifted the lid of the box approximately 3-4 inches for approximately 1.5 s. Following that, the duck’s head propped up to face the infant.
• On the second attempt, the duck lifted the lid of the box 2-3 inches for approximately 2 s, slightly longer than the previous trial. Again, on this attempt, the duck’s head propped up to face the infant.
• On the third attempt, the duck lifted the lid 3–4 inches for approximately 1 s, but did not face the infant following this attempt. In the online supplementary videos (Hamlin et al., 2011), the third attempt differed between helper and hinderer scenarios in both duration of lifting the lid and the approximate height the lid was lifted. However, Hamlin and Wynn (2011) did not describe these variations and so our methods followed the published methods (Hamlin & Wynn, 2011) and kept the actions during the third attempt consistent across puppets.
• On the fourth attempt, the duck lifted the lid 3–4 inches for 1 s, and again faced away from the infant following the attempt.
• Finally, on the fifth attempt, the duck lifted the lid 3–5 inches for approximately 1.5 s. Following the fifth attempt, either a helper or hinderer elephant puppet entered on the side opposite the duck.

#### Helping event puppet show

During the helping events, the helper puppet moved from the side opposite the duck, slid forward, paused, and then was placed face down on the side corner of the box opposite the duck, all of which took approximately 3 s. Together, the elephant and the duck then opened the box lid towards the back of the stage. Next, the duck was placed face down inside the box and grabbed the rattle; this took approximately 2 s. The helper puppet then exited towards the back of the stage in straight line, facing the infant. All action was then halted and the infant’s looking time was measured. After the infant looked away from the stage for 2 s, the curtain was closed.

#### Hindering event puppet show

During hindering events, rather than assisting the duck on its fifth attempt at opening the box, a hinderer elephant puppet slid forward from the side opposite the duck, and then paused at the middle side of the box (the timing of these actions was identical to those in the opening event). However, instead of helping the duck open the box, the hinderer puppet was placed upright on the lid of the box and forcibly slammed the box shut. The duck was placed face down next to the box, and the hinderer puppet was then placed upright next to the side of the box opposite the duck. The hinderer then exited the stage in a straight line, while still facing the infant. All action was then halted, looking time was measured, and the curtain was closed.

#### Choice measure

Parents were instructed to turn their chairs 90 degrees to the right so they were no longer facing the puppet stage, and then to close their eyes until the experimenter told them to open their eyes [2, 5]. Experimenter 2, blind to which puppet was the helper/hinderer, presented the two puppets to the infant, holding each puppet about 25 cm from one another and equidistant from, but initially out of the infant’s reach. Puppets were centered on the infant’ s chest about 30 cm apart and out of the infant’s reach. The infant was next required to look at both puppets, and then look back at Experimenter 2. First, Experimenter 2 said to the infant, “Look!” and slightly shook one puppet until the infant made eye contact with the relevant puppet, and then said, “Look!” and slightly shook the other puppet until the infant made eye contact with it. Finally, Experimenter 2 moved both puppets out of the infant’s line of sight and said “Look!” until the infant looked at the experimenter. Experimenter 2 always shook the puppet on the left side first and the side on which each puppet was presented first was counterbalanced. After the infant made eye contact with Experimenter 2, the puppets were placed approximately 25 cm from one another, equidistant from one another and the infant, but closer to the infant. Experimenter 2 then asked, “Which one would you like to play with?” [2]. Experimenter 2 then repeated the choice measure, but changed the order in which the puppets were presented to the infant. The experimenter conducted the choice measure for a total of five trials. A third experimenter, blind to which puppet was the helper or hinderer, coded the infant’s choice as the first puppet the infant looked at and concurrently touched. To assess interobserver agreement, an independent observer, coder blind to which puppet was the helper or hinderer, later coded infants’ choices by watching a video of the session and independently coding infants’ puppet choices, described in more detail below.

### Interobserver agreement

Because the reliability of the key dependent variable, infant puppet choice, was crucial to accurately interpreting the data, all infant choice trials were video recorded; all in-session infant puppet choices, already coded by two observers, were later coded by a third observer watching the video recordings. Interobserver agreement was calculated for 100% of choice trials within session, and for 25% of the video recorded choice trials. An agreement was scored if both observers identified the same puppet as the one the infant first looked at and concurrently touched on a given trial.

Percent interobserver agreement (IOA) was calculated by dividing the number of agreements by the total number of trials and then multiplying by 100% [21]. Percent agreement for exact duration of looking time in seconds was 75% (range 50–86%); however, agreement was 100% for habituation, the key variable on which the decision to continue or end the habituation trial was made. In-session agreement for 19 of the 20 infants was 96% (range 80–100%). In-session agreement for one infant was not calculated because the infant moved too fast during the choice measure; this infant’s choices were coded from the video. In addition to calculating IOA for this video, we randomly selected four videos (20%) in order to have independent observers code the infant choices. The IOA for those five videos (and 25 choices) was 100%.

### Questionnaire

To assess whether parents’ knowledge of the purpose of the puppet show was associated with infants’ choices, after infants made all five choices, parents were asked to freely respond to the question, “What do you think the puppet show was about?” Parents were scored as having knowledge of the content of the puppet show if they wrote “helping,” “hindering,” “good,” “bad,” or several other similar keywords which were identified as such prior to coding any of the questionnaires (see Table 2 for a complete list of words). Of the 19 parents that responded, 14 (74%) described the puppet show using one or more of these key terms, indicating some knowledge of the purpose of the puppet show (e.g., “a *good* puppet *helped* the duck while a *bad* puppet *hindered* the duck).

**Table 2:** Terms constituting knowledge of the intent of the puppet show (listed in alphabetical order).

Aide	Good	Obstruct
Antagonist(ic)	Help, Helping, Helper	Prevent
Antisocial	Impede	Prosocial
Assist	Interfere	Right
Counteract	Mean	Selfish
Evil	Moral	Wrong
Generous	Nice	

*Note*. These terms were used to code parents’ response to the question, “What do you think the puppet show is about?” Use of any of the above terms was coded as an indication that parents demonstrated knowledge of the purpose of the puppet show.

### Analyses

We conducted several types of analyses. First, we examined the number of infants who chose the helper puppet on the first trial, akin to the results reported by Hamlin and Wynn [2], as well as the number of infants who chose the puppet on the right side on the first trial. Second, we examined the number of infants who chose the helper puppet or the puppet on the right side on the first trial separately for each age group (5–9 month-olds and 10–16 month-olds). Third, we examined the percentage of infants who chose the helper puppet on each trial and calculated conditional probabilities based on these numbers (see description below). Fourth, we examined stability of within-subject choice of the helper puppet across all five trials for all infants and separately for the two age groups. Fifth, we examined within-subject choice stability based on the side on which a puppet was presented. Lastly, we conducted conditional probabilities examining the probability infants would choose the helper (or right side puppet) given they chose the helper (or right side puppet) on the first trial for both the whole group and separated by parents’ knowledge of the purpose of the study.

Between-group comparisons were made using two-tailed binomial and Fisher’s exact test statistical analyses [2]. Conditional probabilities were calculated for infants’ choices on the second through the fifth trial given their responding on the first trial. We chose to calculate the conditional probabilities based on infants’ first choice, as this is the data point most often reported in the literature [1–8]. Conditional probabilities were calculated for the helper puppet and the puppet presented on the right side. For example, the probability that infants selected the helper puppet on the second trial given their responding on the first trial was calculated by taking the number of infants that selected the helper on both the second trial and first trial (e.g. 5) and dividing this by the total number of infants that selected the helper on second trial (e.g., 9), yielding a value (e.g., 0.56) indicating the probability (e.g., 0.56) that an infant would choose the helper puppet two consecutive times during the first and second choice trials. Within-subject analyses were made using visual analysis of graphed data.

## Results

### First puppet choice

**Fig 1:**
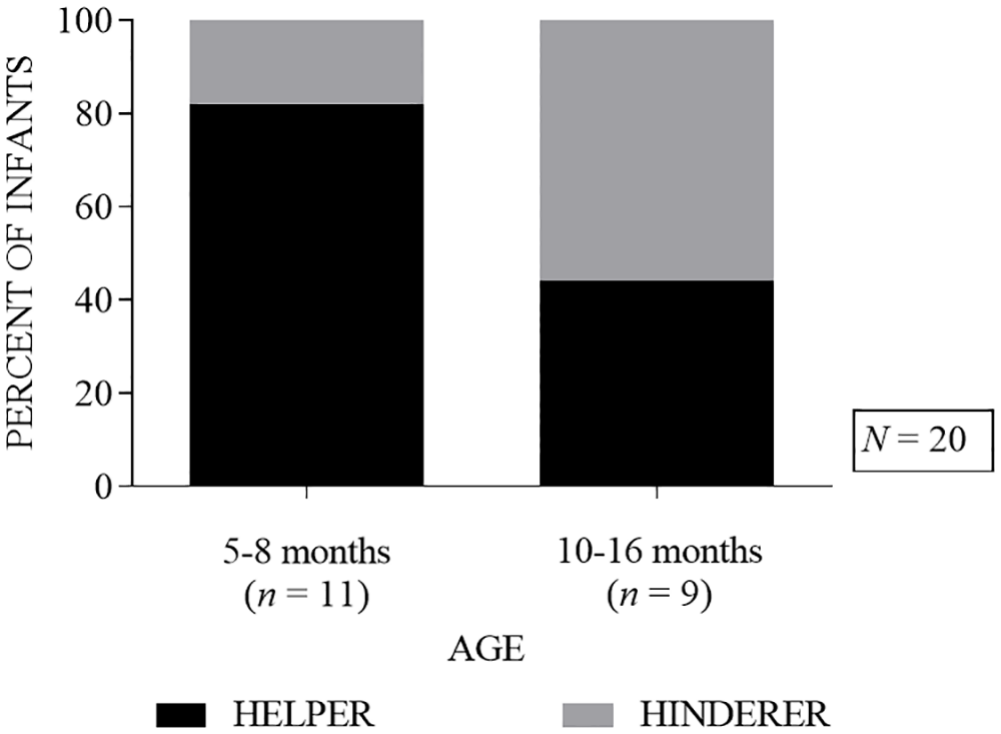
Percent of infants choosing the helper (black bar) or the hinderer (grey bar) on the first trial, separated by age group.

Twenty infants participated in the current investigation. Thirteen (65%) selected the helper puppet on the first trial (*p* = 0.263, binomial test, two-tailed). We also compared results between two age groups, 5–8 month old infants, the same age range used by Hamlin and Wynn [2] and 10–16 month old infants, older than infants in Hamlin and Wynn’s [2] study but similar in age to other comparable studies [1,4]. Among the 5–8 month-old infants (*n* = 11), 9 (82%) selected the helper on the first trial, compared to 4 (44%) of the 10–16 month-old infants (*n* = 9) (see Fig 1); differences between the two age groups were not statistically significant (*p* = .160, Fisher’s exact test).

### First choice based on side

On the first trial, 13 infants (65%) selected the puppet on the right side (*p* = 0.263, binomial test, two-tailed). By age group, 8 (73%) infants in the 5-8-month-old age group (*p* = 0.642, binomial test, two-tailed) and 5 (56%) infants in the 10-16-month-old age group (*p* = 0.246, binomial test, two-tailed) selected the puppet on the right side on the first trial.

### Choice stability across repeated measures

**Fig 2:**
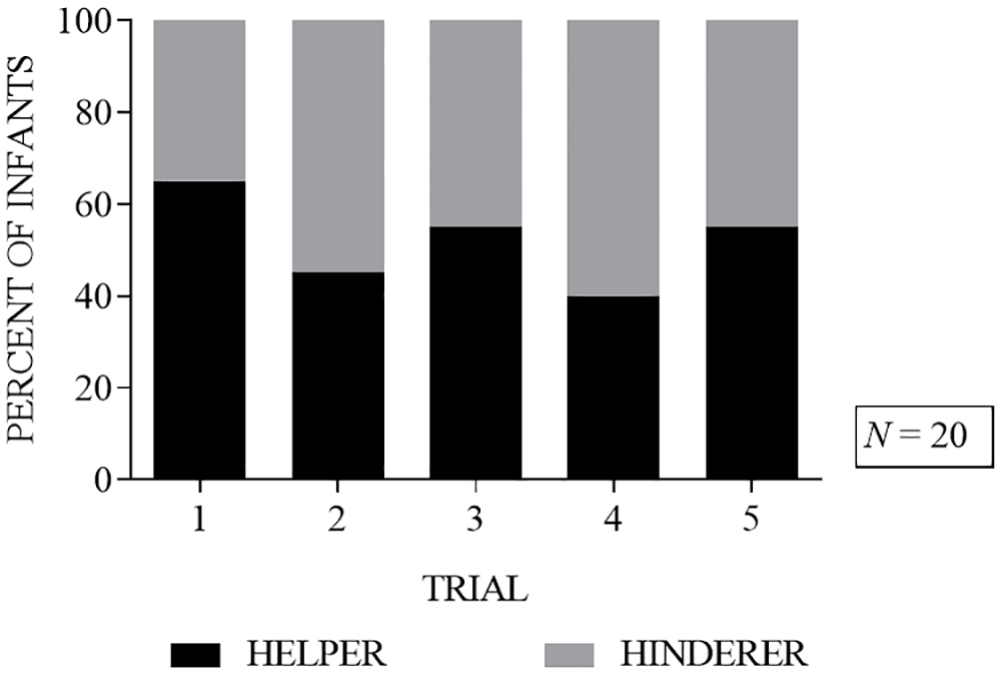
Percent of infants choosing the helper (black bar) or the hinderer (grey bar) in each of the five trials.

**Fig 3:**
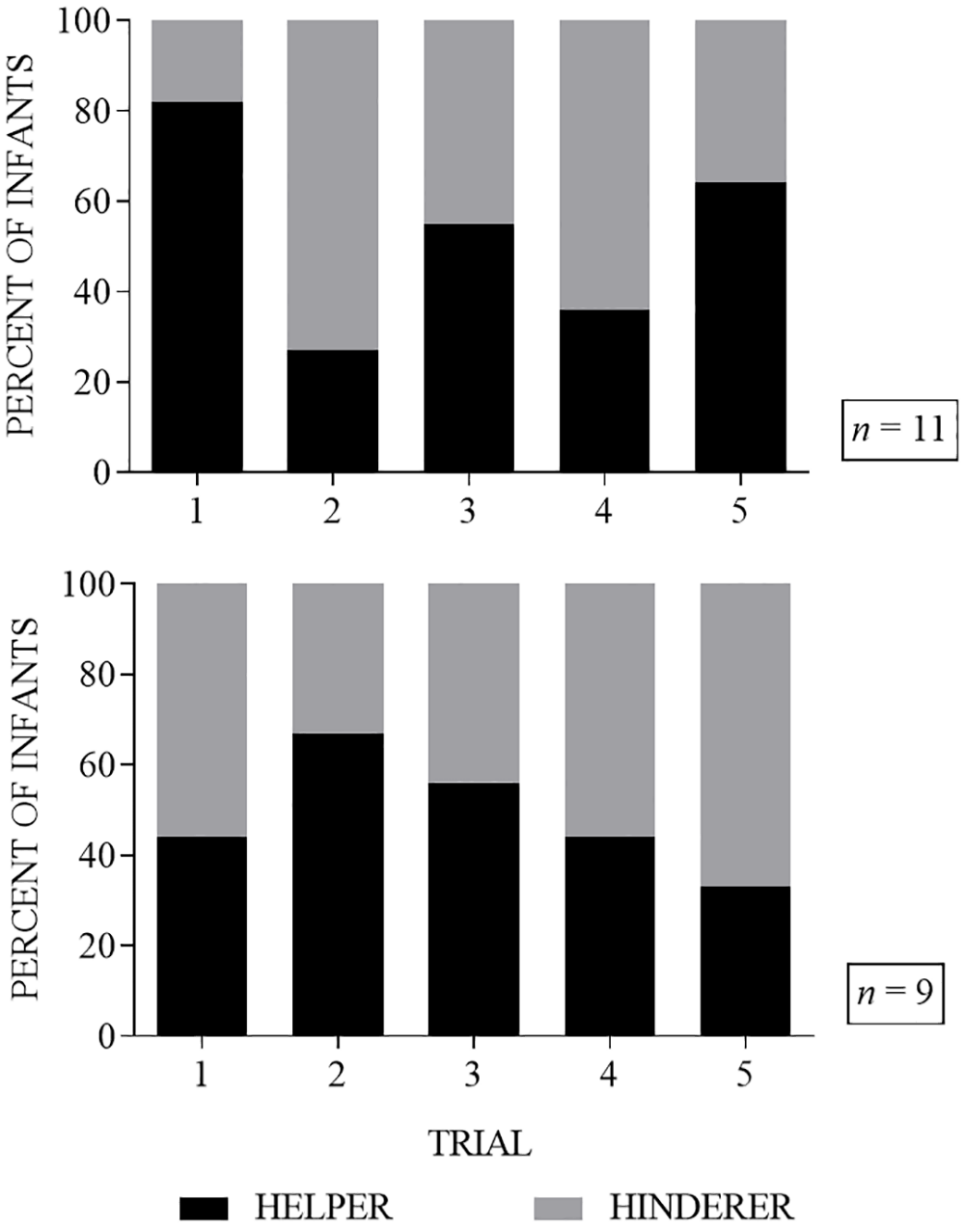
Percent of infants choosing puppets in each of the five trials separated by age group. The top panel consists of infants aged 5–8 months (*n* = 11) and the bottom panel infants age 10–16 months (*n* = 9).

Of the 13 infants who selected the helper puppet on the first trial, only 5 (38%) selected the helper puppet again on the second trial (*p* = 0.5811, binomial test, two-tailed). Among all 20 infants regardless of their choice on the first trial, the helper puppet was selected by 9 infants (45%) on the second trial, 11 infants (55%) on the third trial, 8 infants (40%) on the fourth trial, and 11 infants (55%) on the fifth trial (see Fig 2). Fig 3 shows all 5 trials separated by age group. On the second trial, 3 infants (27%) in the 5-8-month-old age group and 6 infants (67%) in the 10–16 month-old age group selected the helper. On the third trial, 6 infants (55%) in the 5–8 month-old age group and 5 infants (56%) in the 10–16 month-old age group selected the helper. On the forth trial, 4 infants (36%) in the 5-8-month-old age group and 4 infants (44%) in the 10–16 month-old age group selected the helper. On the fifth trial, 7 infants (64%) in the 5–8 month-old age group and 3 infants (33%) in the 10–16 month-old age group selected the helper (see Fig 3).

### Within-subject stability of puppet and side choices across repeated trials

**Fig 4:**
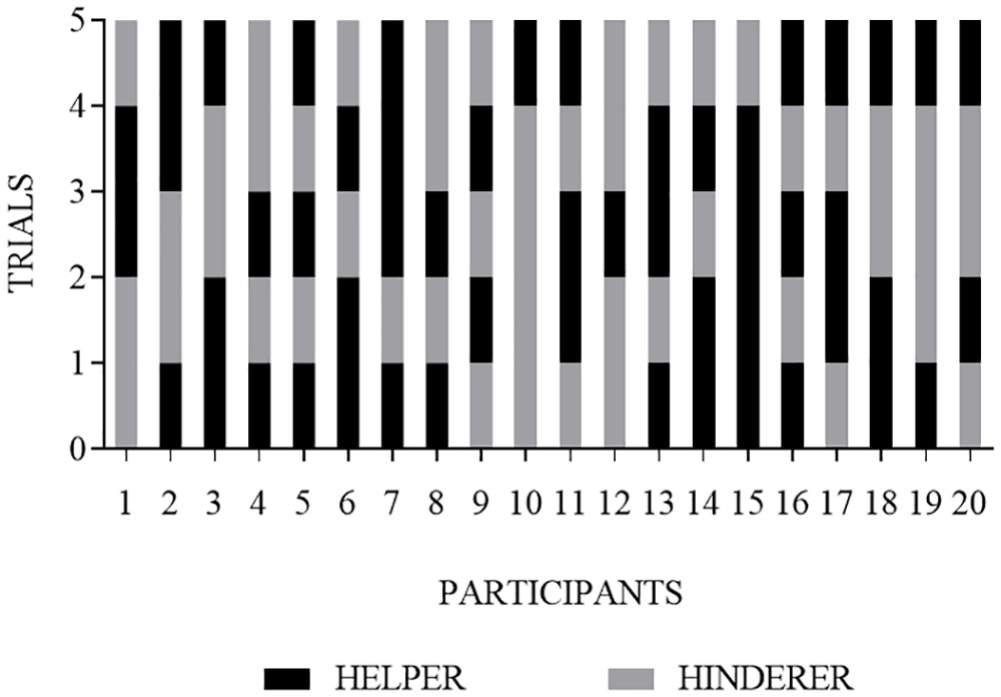
Within-subject analysis of each infant’s choice of the helper (black bar) or the hinderer (grey bar) puppet on each of the five trials. Each bar segment represents an individual infant’s choice for a single trial.

**Fig 5:**
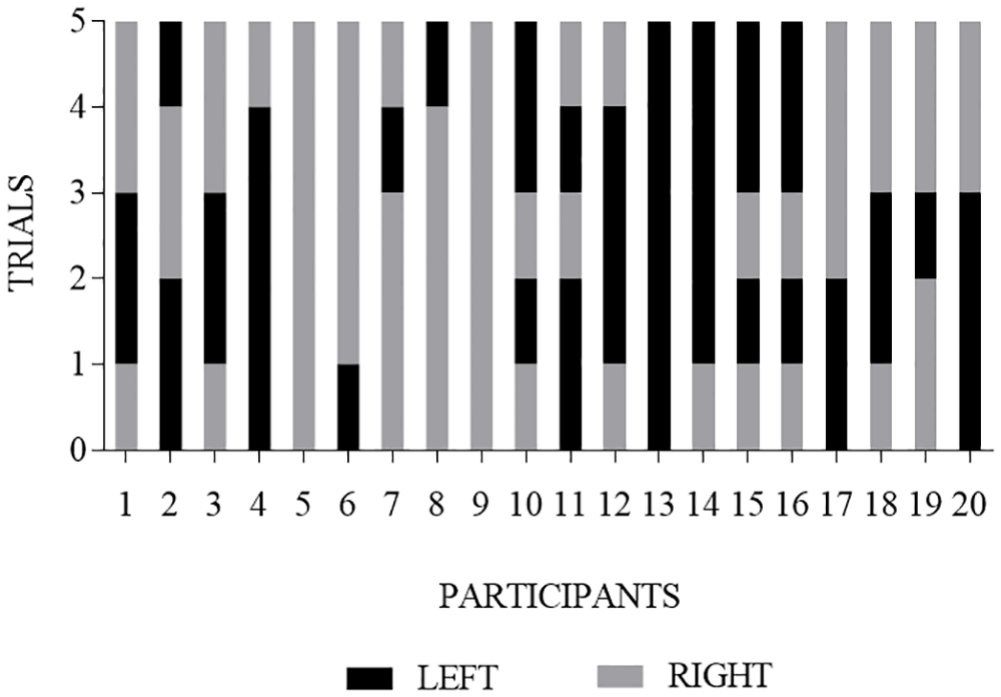
Within-subject analysis of each infant’s choice of the helper (black bar) or the hinderer (grey bar) puppet on each of the five trials separated by age group. Each bar segment represents an individual infant’s choice for a single trial. Along the x-axis, participants are ordered chronologically by age, beginning with the youngest participant on the far left.

No infants chose the helper puppet consistently across all five trials, 2 infants (10%) selected the helper puppet on at least 80% of trials (i.e., at least 4 of 5 trials), and 12 infants (60%) selected the helper puppet on at least 60% of trials (see Fig 4). A similar pattern was observed when examined by age groups (see Fig 5).

**Fig 6:**
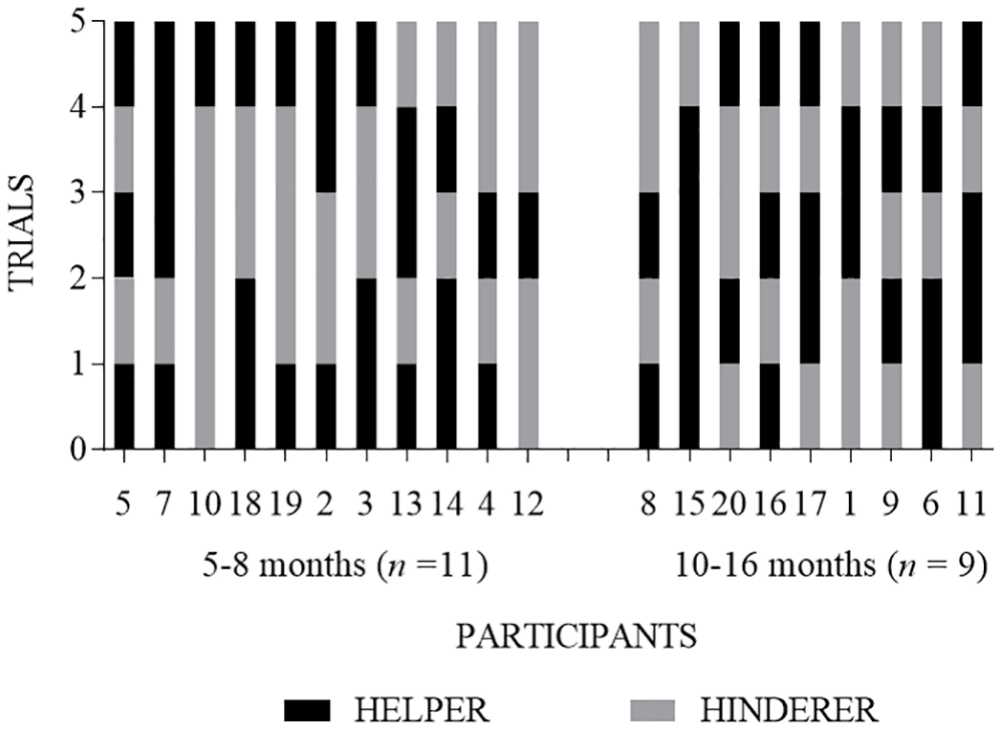
Within-subject analysis of infant choice of the puppet on the left (black bar) or right (grey bar) side on each of the five trials. Each bar segment represents an individual infant’s choice for a single trial.

**Fig 7:**
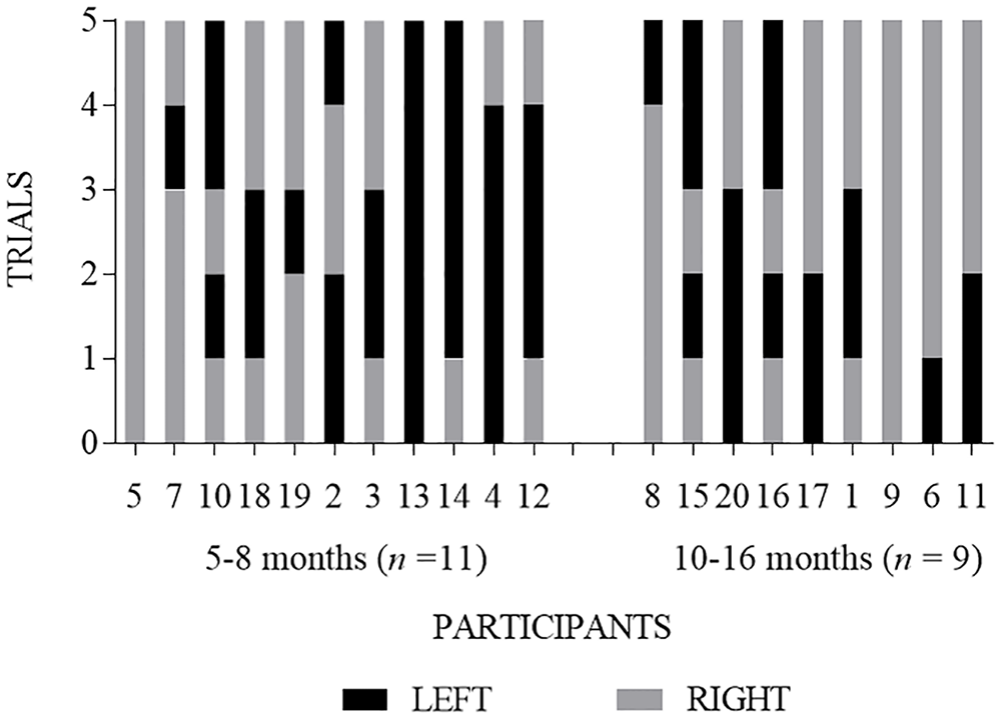
Within-subject analysis of infant choice of the puppet on the left (black bar) or right (grey bar) side on each of the five trials separated by age group. Each bar segment represents an individual infant’s choice for a single trial. Along the x-axis, participants are ordered chronologically by age, beginning with the youngest participant on the far left.

With respect to side, 9 infants (45%) chose the same side on at least 80% of trials, with 3 infants (15%) choosing a puppet on the same side across all 5 trials (i.e., only left or only right side) and 6 infants (35%) choosing a puppet on the same side on at least 80% of trials (see Fig 6). A similar pattern was observed when examined by age groups (see Fig 7).

### Conditional probabilities

Conditional probabilities for infants selecting the same puppet on subsequent trials are reported in Table 3 for both the helper puppet and the right-side puppet. Probabilities ranged from .56 to .75 for selecting the helper puppet and from .62 to .83 for selecting the right-side puppet. Among the 14 infants whose parents demonstrated knowledge of the purpose of the puppet show (e.g., helping and hindering) based on their answers to the questionnaire, 10 infants (71%) chose the helper on the first trial (*p* = 0.180, binomial test, two-tailed); conditional probabilities for these infants choosing the helper puppet on subsequent trials ranged from .40 to .80 (see Table 3).

**Table 3:** Conditional probabilities.

Conditional probability of the same infant choosing the same puppet in Trials 2–5 as chosen in Trial 1			
	Infants who chose the Helper on first trial (*n* = 13)		Infants whose parents showed knowledge of the purpose of the puppet show (*n* = 14)
	Helper Puppet	Right Side Puppet	Helper Puppet
Trial 2	.56 (5/9)	.83 (5/6)	.40 (4/10)
Trial 3	.63 (7/11)	.64 (7/11)	.71 (5/7)
Trial 4	.75 (6/8)	.64 (7/11)	.80 (4/5)
Trial 5	.63 (7/11)	.62 (8/13)	.70 (7/10)

*Note*. The first value (decimal) is the conditional probability and indicates the percent of infants who chose the helper (or right side) puppet on the immediately preceding trial who also chose the helper (or right side) puppet on the current trial. The second value indicates the number of infants who selected the helper (or right side) puppet on both the current and immediately preceding trials (numerator) divided by the number of infants who selected the helper (or right side) puppet in the current trial (denominator). For example, the conditional probability of infants choosing the helper puppet on Trial 2 was calculated by dividing the number of infants who selected the helper puppet on both Trial 2 and Trial 1 (*n* = 5) by the number of infants that selected the helper puppet on Trial 2 (*n* = 9), yielding the value .56 or 56%.

## Discussion

The current study replicated the methods of Hamlin and Wynn’s [2] first experiment. In the current study, 20 infants viewed a puppet show corresponding to that described by Hamlin and Wynn [2]; immediately following the puppet show, each infant was given a choice between two puppets (i.e., the helper or hinderer). We extended Hamlin and Wynn’s [2] methods by having infants make four additional choices after their initial choice. Although our results showed patterns similar to those of Hamlin and Wynn [2] on the first choice trial (65% of infants chose the helper puppet on the first trial), the results were not statistically significant. Moreover, just as many infants (65%) chose the puppet presented on the right side during the first trial, suggesting other variables or factors might be influencing infants’ choices [9, 10, 11]. No infants consistently chose the helper across all 5 trials. On at least 80% of trials (i.e., at least 4 of 5 trials), only 2 infants (10%) chose the helper puppet whereas 9 infants (45%) chose the puppet presented on the right or left side. Conditional probability analyses suggested infants as a group were no more likely to choose the helper puppet than they were to choose the puppet always presented on the right side during the choice measure.

In combination with previously published studies [9, 10, 11], results of the current study add to the growing evidence base against the hypothesis that infants have a robust preference for prosocial others. Robust preferences ought to be stable across multiple trials. Whereas only two infants (10%) chose the helper puppet on at least 80% of trials, nine infants (45%) selected a puppet on the same side on at least 80% of trials. Although it is possible that infants’ first choice reflected their “true” choice, or requiring five consecutive choices confused the infants, the literature examining the most effective manner of identifying individuals’ preferences (e.g., choice), including adults and children with disabilities [22–24] children and infants [25, 26], and even non-human animals [27, 28] suggests choice stability across trials is common and probable. Also, previous research used a similar method for infant choice, whereby infants were asked to make four consecutive choices [4] and researchers equated three of four choices, in any order, with infant preference, although no mention was made of counterbalancing the side on which the foods were presented either within or between subjects. Collectively, findings from these studies [4, 22–28] suggest if the helper puppet was the robustly preferred puppet, most infants would have chosen the helper puppet the majority of trials. Future research should evaluate the degree to which infants’ choices are stable across multiple trials when their preference for an item is already known; this may assist with our knowledge regarding the robustness and reliability of infants’ choices as a measure of preference.

Interpretations of results from the current study must be considered within the context of study limitations. First, given the relatively small sample size, it is difficult to determine if the current study had sufficient power to detect an effect of infant preference. To this point, it is unclear if the effect size of infant preferences is strong, moderate, or weak. If infant preferences are detectable only when using a large sample size, we cannot call their preferences robust; and if several studies employing the same method obtain different results, this also may speak to the robustness (power) of infants’ choices, variability due to use of small samples sizes, and the relative effect size of the phenomenon under investigation [29], at least using the current paradigm. Second, our methods may have varied somewhat from Hamlin and Wynn [2]. For example, based on our reading of their method section and examination of available videos, we chose to lightly shake each puppet to gain the infant’s attention; however, this may not be the same method used by Hamlin and Wynn [2] to gain infants’ attention. Because slight variations in puppet show paradigms appear to produce different results (e.g., [9, 10]), it is important to encourage authors to publish supplemental method sections with extensive details outlining the exact methodology. Moreover, because small methodological variations can have profound influences on study outcomes [30–32], it is important to provide sufficient details about methods and any deviations to help facilitate replications.

In summary, the current investigation addressed limitations of previous research by including both a between-subjects (replication) and a within-subjects (extension) repeated measure of choice that allowed for examination of the stability of infants’ puppet and side choices. The current findings call into question the robustness of infants’ preference for prosocial others given that infants in this study, as well as previous studies [9, 10, 11], demonstrated no clear patterns of preference for the helper puppet, although nearly half of the infants showed a preference for reaching for one side. Studies using this and similar infant choice paradigms should include sufficient methodological detail to facilitate accurate replication and consider incorporating within-subject repeated measures into their designs in order to assess the robustness of infant preferences and reduce the likelihood that findings are spurious or unrepresentative of the phenomenon under investigation.

## Supporting information

S1 Fig Schematic of the experimental arrangement during the choice measure. Notes: Exp refers to Experimenter and the corresponding number indicates which Experimenter; IOA refers to the Experimenter used to calculate interobserver agreement; and, P refers to puppet and the corresponding number indicates which puppet.
(DOCX)

S2 Fig Puppets on the stage from the viewpoint of the infant participant. (DOCX)
