# Supplementary material for: Stability of infants’ preference for prosocial others: Implications for research based on single-choice paradigms
Source: PLoS One. 2017 Jun 2;12(6):e0178818. doi: 10.1371/journal.pone.0178818 (PMC5456381; doi:10.1371/journal.pone.0178818)
Supplement: S1 Fig — Notes: Exp refers to Experimenter and the corresponding number indicates which Experimenter; IOA refers to the Experimenter used to calculate interobserver agreement; and, P refers to puppet and the corresponding number indicates which puppet. (DOCX) [file pone.0178818.s001.docx]

**S1 Fig.** **Schematic of the experimental arrangement during the choice measure.** Exp refers to Experimenter and the corresponding number indicates which Experimenter; IOA refers to the Experimenter used to calculate interobserver agreement; and, P refers to puppet and the corresponding number indicates which puppet.

Exp. 3

Exp. 2 (IOA)

Stage

163 cm

Exp. 1

23cm

P2

P1

Table

Infant

Parent
