# Supplementary figures and images for: Stability of infants’ preference for prosocial others: Implications for research based on single-choice paradigms
Source: PLoS One. 2017 Jun 2;12(6):e0178818. doi: 10.1371/journal.pone.0178818 (PMC5456381; doi:10.1371/journal.pone.0178818)

**S2 Fig. Puppets on the stage from the viewpoint of the infant participant.**


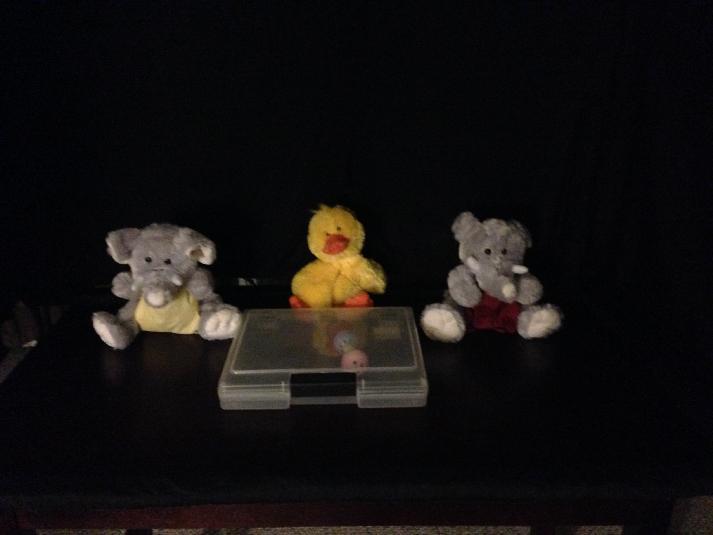

Supplement: S2 Fig — (DOCX) [file pone.0178818.s002.docx]
